# Direct tumor recognition by a human CD4^+^ T-cell subset potently mediates tumor growth inhibition and orchestrates anti-tumor immune responses

**DOI:** 10.1038/srep14896

**Published:** 2015-10-08

**Authors:** Junko Matsuzaki, Takemasa Tsuji, Immanuel F. Luescher, Hiroshi Shiku, Junichi Mineno, Sachiko Okamoto, Lloyd J. Old, Protul Shrikant, Sacha Gnjatic, Kunle Odunsi

**Affiliations:** 1 Center for Immunotherapy, Roswell Park Cancer Institute, Buffalo, NY, USA; 2 Department of Gynecologic Oncology, Roswell Park Cancer Institute, Buffalo, NY, USA; 3 Ludwig Institute for Cancer Research, New York Branch at Memorial Sloan-Kettering Cancer Center, New York, NY, USA; 4 Ludwig Center for Cancer Research, University of Lausanne, Epalinges, Switzerland; 5 Department of Immuno-Gene Therapy, Mie University Graduate School of Medicine, Tsu, Mie, Japan; 6 TAKARA BIO INC, Otsu, Shiga, Japan; 7 Department of Immunology, Roswell Park Cancer Institute, Buffalo, NY, USA; 8 Departments of Immunology, Molecular Pharmacology and Experimental Therapeutics, and Research, Mayo Clinic-Arizona, Scottsdale, AZ, USA; 9 Tisch Cancer Institute, Hematology/Oncology, Immunology, Icahn School of Medicine at Mount Sinai, New York, NY, USA

## Abstract

Tumor antigen-specific CD4^+^ T cells generally orchestrate and regulate immune cells to provide immune surveillance against malignancy. However, activation of antigen-specific CD4^+^ T cells is restricted at local tumor sites where antigen-presenting cells (APCs) are frequently dysfunctional, which can cause rapid exhaustion of anti-tumor immune responses. Herein, we characterize anti-tumor effects of a unique human CD4^+^ helper T-cell subset that directly recognizes the cytoplasmic tumor antigen, NY-ESO-1, presented by MHC class II on cancer cells. Upon direct recognition of cancer cells, tumor-recognizing CD4^+^ T cells (TR-CD4) potently induced IFN-γ-dependent growth arrest in cancer cells. In addition, direct recognition of cancer cells triggers TR-CD4 to provide help to NY-ESO-1-specific CD8^+^ T cells by enhancing cytotoxic activity, and improving viability and proliferation in the absence of APCs. Notably, the TR-CD4 either alone or in collaboration with CD8^+^ T cells significantly inhibited tumor growth *in vivo* in a xenograft model. Finally, retroviral gene-engineering with T cell receptor (TCR) derived from TR-CD4 produced large numbers of functional TR-CD4. These observations provide mechanistic insights into the role of TR-CD4 in tumor immunity, and suggest that approaches to utilize TR-CD4 will augment anti-tumor immune responses for durable therapeutic efficacy in cancer patients.

Activation of tumor antigen-specific T cells is a critical step for tumor regression and/or eradication by the immune system. In this regard, CD4^+^ T lymphocytes have traditionally been described as helpers and regulators of the immune response, and cytotoxic T lymphocyte effector functions have been attributed mostly to CD8^+^ T cells. Despite the inefficient ability of CD4^+^ T cells to directly recognize target cells expressing intracellular proteins such as tumor antigen-expressing cancer cells, a growing body of evidence indicate that tumor antigen-specific CD4^+^ T cells play a pivotal role in orchestrating tumor eradication^1^. The roles of antigen-specific CD4^+^ T cells include provision of help to CD8^+^ T cells during the primary and secondary immune responses, activation/maturation of antigen-presenting cells (APCs), production of cytokines that are essential for differentiation or maintenance of long-lasting T-cell responses, and activation of B cells for the production of tumor antigen-specific antibodies^2^,^3^.

Professional APCs such as dendritic cells play indispensable roles in priming and boosting immune responses at lymphoid organs by cross-presenting antigens, providing co-stimulatory signals, and producing cytokines such as IL-12. Professional APCs are especially important for stimulating antigen-specific CD4^+^ T cells as they are the only cell type that can efficiently cross-present exogenous antigen in the context of MHC-II to CD4^+^ T cells. Tumor antigen-specific CD4^+^ T cells are activated at the local tumor site when tumor-infiltrating APCs capture and cross-present tumor antigens. However, the APCs at the tumor microenvironment are frequently immunosuppressive and lead to unresponsiveness of T cells^4^, which may restrict the activation of CD4^+^ T cells and therefore limit the provision of CD4-help at the tumor microenvironment. An alternative path by which tumor antigen-specific CD4^+^ T cells could overcome the requirement for APCs within the tumor microenvironment is to directly recognize cancer cells. In mouse models, antigen-specific CD4^+^ T cells that directly recognize tumors and exert potent anti-tumor effects have been described^5^,^6^,^7^,^8^. However, antigen-specific TCR transgenic CD4^+^ T cells were used in these model systems, and may not reflect the physiological role of direct tumor recognition by CD4^+^ T cells. Therefore, it is important to understand the role of CD4^+^ T cells that are naturally induced in the tumor-bearing host and directly recognize tumors in the absence of APCs, and test whether they can counteract tumor progression and facilitate anti-tumor immune responses in humans.

Many current tumor vaccine trials aim to simultaneously activate tumor antigen-specific CD4^+^ and CD8^+^ T cells, expecting a synergistic anti-tumor effect. Although simultaneous induction of antigen-specific CD4^+^ and CD8^+^ T cells has been detected in some vaccinated patients^9^,^10^,^11^, their clinical efficacy has been limited. In a previous clinical trial of peptide vaccination aimed at inducing tumor antigen-specific CD8^+^ and CD4^+^ T cells against NY-ESO-1^12^, patients who were HLA-A*02:01+ (A2) and HLA-DPB1*04:01/*04:02+ (DP4) and had NY-ESO-1-expressing ovarian cancer were repeatedly vaccinated with a peptide, NY-ESO-1_157–170_ that contains highly immunogenic epitopes for A2 (NY-ESO-1_157–165_) and DP4 (NY-ESO-1_157–170_). We found that two functionally distinct subsets of NY-ESO-1_157–170_-specific CD4^+^ T cells were expanded after vaccination. While both subsets recognized exogenous NY-ESO-1 protein pulsed on DP4^+^ target cells, only one type recognized target cells that expressed intracellular NY-ESO-1 including cancer cells^13^. Tumor recognition by CD4^+^ T cells was HLA-DP restricted and NY-ESO-1 specific. Mechanistically, we demonstrated that direct tumor recognition by this latter subset (tumor-recognizing CD4^+^ T cells: TR-CD4) requires non-classical MHC class II (MHC-II) antigen-processing pathways such as proteasomal degradation and transporter-associated with antigen-processing mediated peptide transport, that are typically involved in the MHC class I (MHC-I) presentation, and endosomal recycling. TR-CD4 has the ability to recognize short 8–9-mer NY-ESO-1 peptides (NY-ESO-1_161–168_ or NY-ESO-1_161–169_). Importantly, TR-CD4 recognized fresh ovarian cancer cells in ovarian tumor specimens^13^. In the present study, we investigate anti-tumor functions of TR-CD4 and demonstrate that direct cognate interaction between this subset of human CD4^+^ T helper cells (TR-CD4) and cancer cells efficiently induce growth arrest in cancer cells and potently provide help to cognate tumor antigen-specific CD8^+^ T cells in an APC-independent fashion, resulting in significant anti-tumor activity both *in vitro* and *in vivo*. Finally, we showed that activated T cells that were engineered to express T cell receptor (TCR) from TR-CD4 also directly recognized tumor targets. Thus, our work is unique in showing that human tumor-recognizing CD4^+^ T cells have an important role in controlling tumor progression. These results provide rationale for adoptive T cell therapy trials testing therapeutic efficacy of TR-CD4 alone or in combination with TCR gene-engineered CD8^+^ T cells.

## Results

### Inhibition of cancer cell growth by tumor-recognizing CD4^+^ T cells

**Figure 1:**
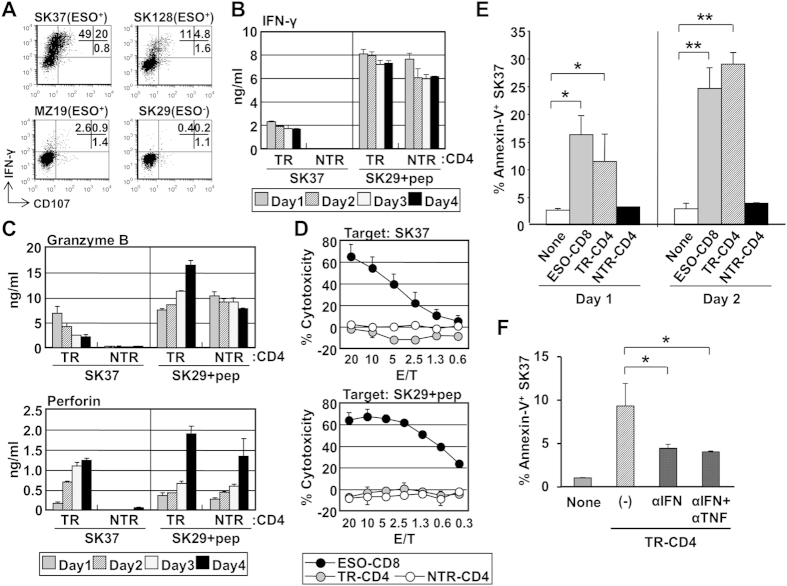
Functional characterization of TR-CD4. (**A**) Recognition of HLA-DP4^+^NY-ESO-1^+^ SK-MEL-37 (SK37), SK-MEL-128 (SK128) or MZ-MEL-19 (MZ19), or HLA-DP4^+^NY-ESO-1^−^ SK-MEL-29 (SK29) by TR-CD4 was tested by cell surface CD107 and intracellular IFN-γ-staining. Numbers indicate the percentages in three quadrants. (**B**,**C**) TR-CD4 and NTR-CD4 were co-cultured with SK37 or NY-ESO-1_157–170_-pulsed SK29 and supernatant was collected every 24 hours for 4 days. IFN-γ (**B**), granzyme B and perforin (**C**) levels in the supernatant measured by ELISA are shown. Background production against unpulsed SK29 was below the detection limit (4 pg/ml). (**D**) Cytotoxic activity of ESO-CD8, TR-CD4 and NTR-CD4 against SK37 or peptide-pulsed SK29 was determined by 14–16-hour CFSE-based cytotoxicity assays. NY-ESO-1_157–165_ or NY-ESO-1_157–170_ was pulsed on SK29 for ESO-CD8 or CD4^+^ T cells, respectively. (**E**) Apoptosis of SK37 after co-culturing with NY-ESO-1-specific T cells was evaluated by staining of annexin-V. SK37 was co-cultured with ESO-CD8, TR-CD4 or NTR-CD4. At day 1 and day 2 of co-culture, cells were harvested and stained with annexin-V-specific antibody. (**F**) SK37 was co-cultured with TR-CD4 in the presence or absence of neutralizing antibodies against IFN-γ (αIFN) and TNF-α (αTNF). At day 3, apoptotic cell death of SK37 was determined by annexin-V staining. Annexin-V-expression on SK37 gated by FSC/SSC pattern was evaluated by flow cytometry.

As reported previously, repeated vaccinations with DP4-binding NY-ESO-1_157–170_ peptide, which encompassed A2-binding NY-ESO-1_157–165_ epitope, could induce robust CD4^+^ T-cell responses in addition to NY-ESO-1-specific CD8^+^ T cells and antibodies^12^. Unexpectedly from the view of classical antigen presentation pathways, a minor fraction of DP4-restricted CD4^+^ T cells responded to NY-ESO-1^+^A2^+^DP4^+^ SK-MEL-37 (SK37). To further characterize the functional differences between TR-CD4 and non-tumor-recognizing CD4^+^ T cells (NTR-CD4) in detail, we established NY-ESO-1-specific and DP4-restricted TR-CD4 and NTR-CD4 clones^13^. Using the same clones used for the comparative mechanistic analysis in our previous report^13^, we first tested tumor-recognizing ability of TR-CD4 against several DP4^+^ cancer cell lines, in the absence of APCs. TR-CD4 selectively recognized NY-ESO-1^+^ but not NY-ESO-1^−^ DP4^+^ melanoma cell lines (Fig. 1A). Because we could not identify any ovarian cancer cell line in our cell bank that naturally expresses both the appropriate cell surface MHC-II and intracellular NY-ESO-1, we generated two ovarian cancer lines with these features. Two DP4^+^ ovarian cancer cell lines (OV2774 and OVCAR-5) were transduced with a self-inactivating bicistronic retroviral vector encoding class II transactivator (CIITA), which induced cell surface MHC-II expression, and NY-ESO-1 or control human sperm protein 17 (Sp17) gene (Supplementary Fig. S1A). TR-CD4 recognized both OV2774 and OVCAR-5 that expressed NY-ESO-1/CIITA but not Sp17/CIITA. After CIITA-expressing ovarian cancer cell lines were pulsed with NY-ESO-1_157–170_ peptide, they were efficiently recognized by both NTR-CD4 as well as TR-CD4 (Supplementary Fig. S1B).

Because SK37 was most strongly recognized, it was used as the prototypic NY-ESO-1 and MHC-II-expressing target cell in subsequent experiments. Whereas both TR-CD4 and NTR-CD4 similarly responded to NY-ESO-1_157–170_ peptide pulsed on NY-ESO-1^−^A2^+^DP4^+^ SK-MEL-29 (SK29), only TR-CD4 recognized NY-ESO-1-expressing SK37 (Fig. 1B). Because several murine studies demonstrated that tumor-reactive CD4^+^ T cell acquires cytotoxic capacity against tumors^5^,^6^, we assessed the direct cancer cell lytic capacity of TR-CD4. While TR-CD4 and NTR-CD4 similarly released perforin and granzyme B upon stimulation with NY-ESO-1_157–170_ peptide-pulsed SK29, only TR-CD4 produced cytolytic molecules against peptide-unpulsed SK37 (Fig. 1C). However, in contrast to the strong cytolytic activity by a control A2-restricted NY-ESO-1-specific CD8^+^ T cells (ESO-CD8), neither TR-CD4 nor NTR-CD4 were cytotoxic against cancer cells, even when the cancer cells were pulsed with peptide (Fig. 1D). Despite the fact that TR-CD4 did not show immediate cytotoxicity, we observed that SK37 underwent apoptosis after 1–2 days of co-culture with TR-CD4 but not NTR-CD4 to a similar level as observed with ESO-CD8 (Fig. 1E), raising the possibility that TR-CD4 may mediate delayed cancer cell death rather than immediate cytotoxicity. Consistent with the strong IFN-γ production from NTR-CD4 and TR-CD4 against NY-ESO-1_157–170_ peptide-pulsed SK29, both NTR-CD4 and TR-CD4 induced apoptosis on peptide-pulsed but not peptide-unpulsed SK29 (Supplementary Fig. S2A).

**Figure 2:**
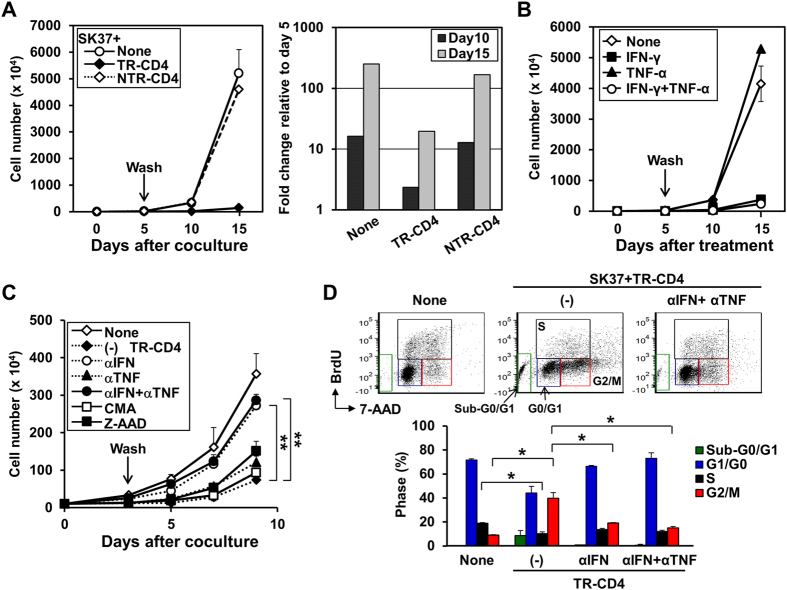
Long-term growth arrest of cancer cells mediated by TR-CD4. (**A**) SK37 was co-cultured with or without TR-CD4 or NTR-CD4 for 5 days. Non-adherent T cells were removed by repeated rinses using culture medium and adherent SK37 were further cultured for 10 days. Cell numbers were determined by trypan blue exclusion assays. Fold expansion was calculated by dividing the cell number on day 10 or 15 by the cell number on day 5. (**B**) SK37 was cultured with recombinant IFN-γ and/or TNF-α for 5 days. Adherent SK37 were rinsed and cultured in the medium without cytokines for 10 days. Cell numbers were determined by trypan blue exclusion assays. (**C**) SK37 was co-cultured with TR-CD4 in the presence or absence of neutralizing antibodies against IFN-γ (αIFN) or TNF-α (αTNF), or inhibitors for perforin (concanamycin A: CMA) or granzyme B (benzyloxycarbonyl-Ala-Ala-Asp-chloromethylketone: Z-AAD) for 3 days. Non-adherent T cells were removed by repeated rinses and adherent SK37 were further cultured. Cell numbers were determined at the indicated days after the culture. **P < 0.01 at day 9 by Student’s *t*-test. (**D**) SK37 was co-cultured with TR-CD4 in the presence or absence of αIFN and αTNF. At day 3 of co-culture, cell cycle of SK37 was analyzed by staining with anti-BrdU antibody and 7-AAD.

Recently, temporal treatment with the combination of CD4-derived cytokines, IFN-γ and TNF-α, was reported to induce long-term growth arrest in cancer cell lines^14^. We therefore investigated whether these cytokines produced from TR-CD4 induced long-term growth arrest in SK37. TR-CD4 secreted both IFN-γ and TNF-α when cocultured with SK37 (Fig. 1B, Supplementary Fig. S3A). SK37 expressed the receptors for IFN-γ and to a lesser extent for TNF-α (Supplementary Fig. S3B). To investigate the long-term effect of the Th1-cytokines on SK37 cell growth, SK37 was co-cultured with NY-ESO-1-specific CD4^+^ T cells or recombinant cytokines for 5 days and non-adherent T cells or cytokines were washed out. Co-culture with TR-CD4 but not NTR-CD4 for 5 days substantially reduced the growth of SK37 (Fig. 2A). In contrast, peptide-pulsed SK29 growth was similarly inhibited by TR-CD4 and NTR-CD4 (Supplementary Fig. S2B). A similar degree of growth arrest on SK37 was observed by treatment with recombinant IFN-γ (Fig. 2B and Supplementary Fig. S3C), demonstrating that IFN-γ signaling alone induces long-term cell growth arrest in SK37. Similar durable cell growth inhibition by TR-CD4 or IFN-γ was also induced in SK-MEL-128, another NY-ESO-1^+^ DP4^+^ cell line (Fig. 1A and Supplementary Fig. S4). The marginal effect of TNF-α on the SK37 and SK-MEL-128 growth can be attributed to the low expression of TNF-α receptor on these cell lines (Supplementary Figs S3B and S4C). Moreover, the growth arrest induced by TR-CD4 was abrogated by neutralizing antibody against IFN-γ but not by anti-TNF-α mAb or inhibitors for perforin (concanamycin A: CMA) or granzyme B (Z-Ala-Ala-Asp-Chloromethyl Ketone: Z-AAD), indicating the critical role of TR-CD4-derived IFN-γ in inducing growth arrest (Fig. 2C). Since TR-CD4 also induced apoptotic cell death in SK37 (Fig. 1E), we assessed whether anti-IFN-γ, or the combination of anti-IFN-γ and anti-TNF-α mAb treatment reduces apoptotic cell death. Similar to their effects on cell growth, these antibodies abrogated TR-CD4-mediated apoptosis (Fig. 1F). In addition, TR-CD4 produced IFN-γ and TNF-α to levels that additively induce apoptosis in SK37 (Supplementary Fig. S3E).

Analysis of cell cycle phases in SK37 revealed accumulation in the G2/M phase after co-culture with TR-CD4 in addition to the sub-G0/G1 apoptotic population, and decrease in the S phase compared to untreated SK37 (Fig. 2D). Anti-IFN-γ treatment restored G2/M arrest caused by TR-CD4, while the effect by anti-TNF-α mAb was marginal (Fig. 2D). The G2/M arrest induced by TR-CD4 was recapitulated by treatment with IFN-γ (Supplementary Fig. S3D). Thus, IFN-γ produced from TR-CD4 upon direct recognition of cancer cells, plays a critical role in mediating growth arrest in cancer cells by inducing apoptosis and cell cycle arrest in the G2/M phase.

### TR-CD4 enhances CD8^+^ T cell function *in vitro* and *in vivo*

**Figure 3:**
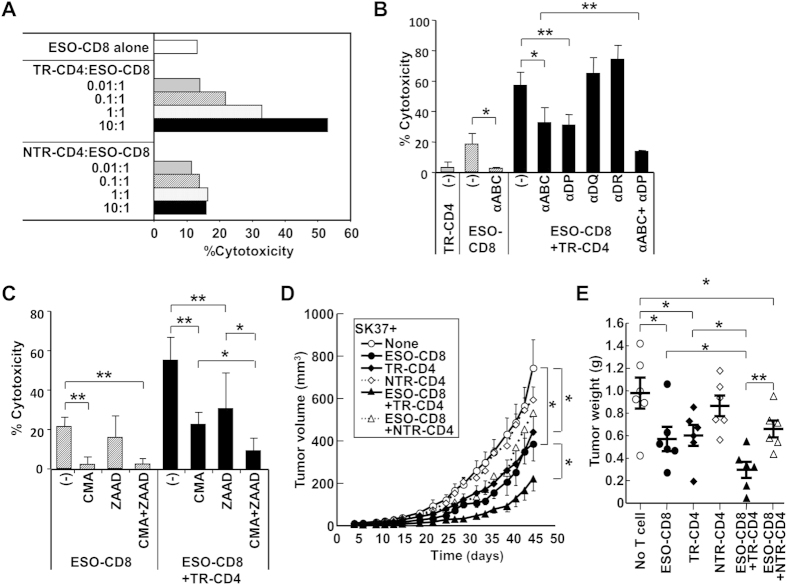
Enhancement of CD8^+^ T cell cytotoxicity via direct tumor recognition by TR-CD4. (**A**) SK37 was co-cultured with ESO-CD8 at 1:2 ratio in the presence or absence of indicated numbers of TR-CD4 or NTR-CD4. Cytotoxicity was evaluated by CFSE-based cytotoxicity assays. (**B**) Effect of blocking antibodies against HLA on cytotoxicity. SK37 was preincubated with anti-HLA-class I (αABC), HLA-DP (αDP), HLA-DQ (αDQ), or HLA-DR (αDR) before mixing with TR-CD4 and/or ESO-CD8 in CFSE-based cytotoxicity assays. (**C**) Mechanism of cytotoxic activity by ESO-CD8 in the presence or absence of TR-CD4. Inhibitors for perforin (CMA at 0.1 nM) and/or granzyme B (Z-AAD at 100 μM)-mediated cytotoxicity was added during CFSE-based cytotoxicity assays. (**D**,**E**) *In vivo* anti-tumor effect by ESO-CD8 and/or CD4^+^ T cells. SK37 was inoculated into SCID mice (n = 6 per group) together with or without ESO-CD8 and/or TR-CD4 or NTR-CD4. D, Tumor size was measured every 2–3 days during the observation period (mean ± SEM). *P < 0.05, **P < 0.01 at day 45 by Student’s *t*-test. E, The tumor was excised and weighted at day 45 after inoculation (mean ± SEM).

Provision of CD4-help generally requires MHC-II antigen cross-presentation by APCs. To investigate whether direct tumor recognition by TR-CD4 could bypass the requirement for APCs to enhance the function of CD8^+^ T cells, ESO-CD8 was co-cultured with SK37 in the presence of TR-CD4 or NTR-CD4. Although TR-CD4 did not show any immediate cytotoxicity *in vitro* (Fig. 1D), TR-CD4 but not NTR-CD4 significantly enhanced CD8^+^ T cell cytotoxicity in a dose-dependent fashion (Fig. 3A). Blockade of HLA-DP abrogated the enhancement of CD8^+^ T-cell cytotoxicity, indicating the requirement of direct tumor recognition by TR-CD4. The combination of HLA-DP and HLA-ABC neutralizing antibodies more efficiently inhibited TR-CD4-augemented cytotoxicity (Fig. 3B). Cytotoxic activity by ESO-CD8 alone is significantly inhibited only by the inhibitor for perforin-dependent pathway while the effect of granzyme-inhibitor was profound in the presence of TR-CD4 (Fig. 3C).

Anti-tumor effect of CD4^+^ T cells alone or in combination with CD8^+^ T cells was further investigated *in vivo* in a xenograft mouse model. In this model, SK37 was inoculated subcutaneously in SCID mice together with NY-ESO-1-specific T cells and tumor growth was monitored. ESO-CD8 alone significantly delayed tumor growth. TR-CD4 but not NTR-CD4 showed significant *in vivo* tumor growth inhibition that was similar to ESO-CD8 (Fig. 3D,E). As expected, mice receiving both ESO-CD8 and TR-CD4 showed the most effective anti-tumor effects. *In vivo* anti-tumor effect by ESO-CD8 and/or TR-CD4 was not observed against NY-ESO-1-negative SK29, demonstrating antigen-specificity in this model (Supplementary Fig. S5).

### Mechanisms of TR-CD4 promotion of antitumor effects of CD8^+^ T cells

**Figure 4:**
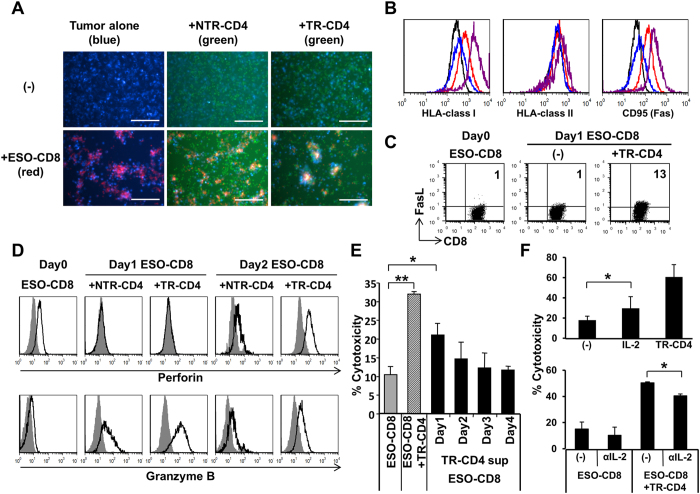
Mechanism of the enhancement of CD8^+^ T cell cytotoxicity by direct tumor recognition by TR-CD4. (**A**) Interaction of cancer cells and T cells. SK37 (blue), ESO-CD8 (red) and TR-CD4 or NTR-CD4 (green) were labeled before co-culture. Fluorescent image was recorded 20–24 hours after co-culture at 200× magnification. Scale bars indicate 200 μm. (**B**) Expression of cell surface molecules on SK37 after co-culture with ESO-CD8 (red), TR-CD4 (blue), or ESO-CD8+TR-CD4 (purple) for 24 hours. Baseline expression by SK37 alone is shown by black solid line. (**C**) Fas-ligand (FasL) expression on CD8^+^ T cells. ESO-CD8 was stimulated with SK37 in the presence or absence of TR-CD4. FasL expression on ESO-CD8 was evaluated by flow cytometry before (Day 0) and after stimulation (Day 1). (**D**) Expression of cytotoxic molecules in CD8^+^ T cells. ESO-CD8 was stimulated by SK37 in the presence of TR-CD4 or NTR-CD4. Intracellular perforin and granzyme B expression before (day 0) and after (day 1 and day 2) stimulation were measured by flow cytometry. Shaded histogram shows the background staining by the isotype control antibody. (**E**) Effect of TR-CD4-derived soluble factors on CD8^+^ T cell cytotoxicity. ESO-CD8 was stimulated with SK37 in the presence or absence of TR-CD4 or culture supernatant of TR-CD4 stimulated with SK37 harvested at days 1–4. (**F**) Effect of IL-2 on CD8^+^ T cell cytotoxic activity. Recombinant IL-2 (upper) or neutralizing mAb against IL-2 (αIL-2) (lower) was added during CFSE-based cytotoxicity assays. Cytotoxicity was measured by CFSE-based assays.

Microscopic analyses showed that ESO-CD8 but not TR-CD4 formed clusters with cancer cells (Fig. 4A). TR-CD4 participated in the cluster formation when ESO-CD8 was present. The formation of clusters that are composed of cancer cells, ESO-CD8, and TR-CD4 is considered to facilitate the close interaction between these cell types leading to efficient elimination of cancer cells. Such reinforcement of clustering was not observed when cancer cells were co-cultured with ESO-CD8 and NTR-CD4. Remarkably, upon co-culture of ESO-CD8 with TR-CD4, MHC-I and Fas expression on SK37 were significantly increased compared with co-cultures of SK37 and ESO-CD8 alone (Fig. 4B). We found that treatment of SK37 by recombinant IFN-γ but not TNF-α partially reproduced the upregulation of MHC-I and Fas (data not shown). However, ESO-CD8 more efficiently upregulated MHC-I and Fas although they produced less IFN-γ compared with TR-CD4 (Supplementary Fig. S6B). In addition, neutralization by anti-IFN-γ antibody did not inhibit the upregulation (data not shown). These results indicated that other T cell-derived molecule(s) upregulate MHC-I and Fas on cancer cells.

By live cell imaging, whereas ESO-CD8 stably attached to cancer cells until cancer cell lysis, TR-CD4 constantly moved between cancer cells (Supplementary Movie S1), potentially explaining the difference in the cytotoxic activity between these cell types. It is possible that the brief TR-CD4 contact with cancer cells alters the microenvironment by producing cytokines and upregulating MHC-I and Fas expression on cancer cells.

Furthermore, cell surface Fas ligand (FasL), cytoplasmic perforin and granzyme B expression by ESO-CD8 were increased when co-cultured with TR-CD4 (Fig. 4C,D). The increase of granzyme B expression in ESO-CD8 is consistent with the profound inhibition of cytotoxicity by granzyme inhibitor in the presence of TR-CD4 (Fig. 3C). In contrast, TR-CD4 did not accumulate cytolytic molecules in the cytoplasm (data not shown). Instead, these lytic molecules were detected at higher levels in the supernatant of TR-CD4 than that of ESO-CD8 (Supplementary Fig. S6A). Although upregulation of Fas on SK37 and FasL on ESO-CD8 could contribute to the enhanced cytotoxicity, blocking Fas/FasL interaction by antibody showed minimal effect (Supplementary Fig. S7A). We therefore examined the sensitivity of SK37 to Fas-mediated cell death using anti-Fas mAb and cross-linking recombinant Protein G. In comparison with Fas-sensitive Jurkat cells, Fas cross-linking did not affect SK37 cell viability and only marginally induced early apoptosis (Supplementary Fig. S7B and S7C). From these observations, although TR-CD4-mediated upregulation of Fas on SK37 and FasL on ESO-CD8, this pathway played a minimal role in the enhancement of cytotoxicity in our experimental setting using SK37.

To further examine the potential mechanism(s) by which direct tumor recognition by TR-CD4 enhance CD8^+^ T-cell cytotoxicity, the effect of TR-CD4-derived soluble factors on the cytotoxicity of ESO-CD8 was investigated. Addition of supernatant from co-culture of TR-CD4 and SK37 significantly enhanced the cytotoxicity of ESO-CD8 (Fig. 4E). The strongest enhancement was observed with day 1 supernatant of SK37-stimulated TR-CD4 and decreased thereafter. Measurement of cytokine levels in the supernatant of TR-CD4 showed different kinetics of cytokine production (Supplementary Fig. S6A and S6B). Among measured cytokines, IL-2 level was exclusively detected in day 1 culture supernatant and rapidly decreased. The role of TR-CD4-derived IL-2 in the enhancement of ESO-CD8-mediated cytotoxicity was supported by observations that the addition of recombinant IL-2 partially enhanced ESO-CD8 cytotoxicity, and treatment with anti-IL-2 neutralizing antibody partially abrogated the cytotoxicity enhancement by TR-CD4 (Fig. 4F). Thus, IL-2 production from TR-CD4 after direct tumor recognition was considered to be one of soluble factors that enhanced CD8^+^ T cell cytotoxicity^15^. However, the effect of exogenous IL-2 and blocking anti-IL-2 antibody was modest, indicating additional cell-cell contact-dependent and/or soluble factors are involved in the TR-CD4-mediated enhancement of CD8^+^ T-cell functions. Because CD4^+^ T cells enhance the function of CD8^+^ T cells through CD40-CD40L and CD70-CD27 interactions in the presence of APCs^16^,^17^, the effect of these interactions on the enhancement of ESO-CD8-mediated cytotoxicity by TR-CD4 was examined. However, blockade of CD40-CD40L and CD27-CD70 using antibodies (anti-CD40 antibody: clone 5C3 from eBioscience, and anti-CD27 antibody: clone LG.3A10 from eBioscience) showed no effect on the cytotoxicity of ESO-CD8-mediated cytotoxicity and the enhancement by TR-CD4 (data not shown). Future additional experiments will be required to elucidate the IL-2-independent mechanism(s) for TR-CD4-mediated enhancement of CD8^+^ T-cell functions.

**Figure 5:**
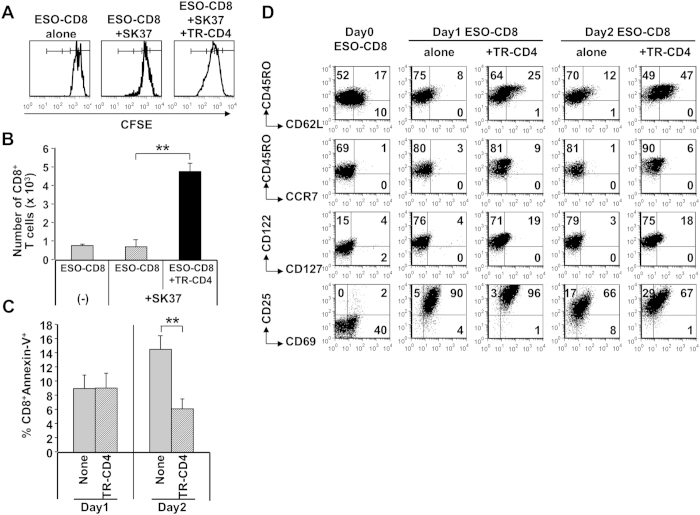
Promotion of CD8^+^ T cell survival by CD4^+^ T cell help via direct tumor recognition. (**A**) CFSE-labeled ESO-CD8 was stimulated with SK37 in the presence or absence of TR-CD4. Unstimulated ESO-CD8 was cultured as a negative control. At day 4, CFSE-staining intensities were measured by flow cytometry to evaluate ESO-CD8 proliferation. Percentages of cells in each division were as follows. ESO-CD8 alone: 1 × divided = 54%, 2 × divided = 0%, 3 × divided = 0%. ESO-CD8+SK37: 1 × divided = 75%, 2 × divided = 9%, 3 × divided = 2%. ESO-CD8+SK37+TR-CD4: 1 × divided = 42%, 2 × divided = 40%, 3 × divided = 16%. (**B**) Cell number was determined by trypan blue exclusion assays combined with CD8^+^ T cell staining by flow cytometry at day 4. (**C**) ESO-CD8 was co-cultured with or without TR-CD4. At day 1 and day 2, apoptotic cell death of ESO-CD8 was determined by annexin-V staining by flow cytometry. (**D**) Phenotype of CD8^+^ T cells stimulated by SK37 with or without help by TR-CD4 via direct tumor recognition. ESO-CD8 was stimulated by SK37 in the presence or absence of TR-CD4. Cell surface expression of indicated molecules on ESO-CD8 were evaluated by flow cytometry before (day 0) and after (day 1 and day 2) stimulation.

Next, we investigated whether TR-CD4, through direct recognition of cancer cells, could enhance proliferation and survival of CD8^+^ T cells, which is one of the important functions of CD4^+^ T cells. ESO-CD8 stimulated with SK37 in the absence of TR-CD4 did not proliferate more than once (Fig. 5A). In sharp contrast, co-culturing with TR-CD4 increased proliferation and reduced apoptosis of ESO-CD8, resulting in significantly greater numbers of ESO-CD8 in co-culture (Fig. 5A–C). In addition, although ESO-CD8 similarly expressed CD25, CD122 and CD69 activation markers when stimulated by SK37 with or without TR-CD4, ESO-CD8 stimulated together with TR-CD4 showed upregulation of central memory differentiation markers such as CD62L, CD127 and modest increase of CCR7 (Fig. 5D). Therefore, TR-CD4 promoted survival and proliferation of tumor-specific CD8^+^ T cells through direct recognition of cancer cells, which could support long-term anti-tumor effect of CD8^+^ T cells at the local tumor site.

### Recognition of cancer cells by TR-CD4 TCR transduced cells

Our *in vitro* and *in vivo* observations indicate that TR-CD4, especially in combination with tumor antigen-specific CD8^+^ T cells, show potent anti-tumor effects. Therefore, we reasoned that generation of large numbers of TR-CD4 could be a promising strategy for efficient therapeutic treatment of cancer patients. However, specific activation of TR-CD4 by vaccination or efficient *in vitro* expansion of TR-CD4 for adoptive T cell therapy may be difficult because of their low frequency. Instead, large numbers of clinically applicable antigen-specific T cells could be generated by gene-engineering with tumor antigen-specific T cell receptor (TCR) gene. TCR gene-engineering strategy has been successfully applied to generate tumor antigen-specific MHC-I restricted T cells^18^,^19^. However, therapeutic anti-tumor effects of MHC-II-restricted tumor-recognizing T cells have not been tested in clinical trials except for a single report that demonstrated that adoptive transfer of NY-ESO-1-specific CD4^+^ T cell clone in a melanoma patient led to complete remission^20^. As DP4-restricted NY-ESO-1_157–170_-specific CD4^+^ T cell clones were used in this trial^20^, we reasoned that the complete remission could be mediated by direct tumor recognition attributes of these CD4^+^ T cell clones. Thus, we investigated whether direct MHC-II-restricted tumor recognition by our TR-CD4 is solely mediated by their TCR and whether large numbers of TR-CD4 could be generated by gene-engineering with tumor-recognizing MHC-II-restricted TCR.

**Figure 6:**
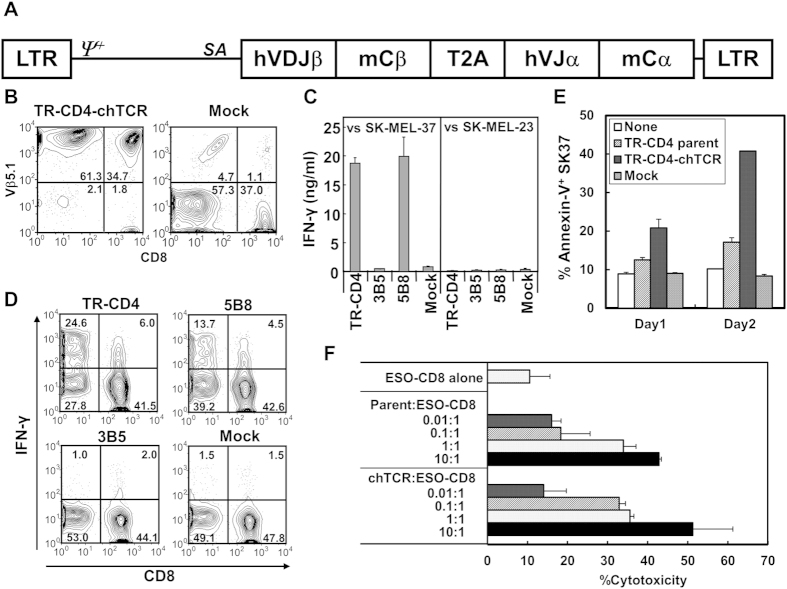
Generation of MHC-II-restricted tumor-recognizing T cells by retroviral TCR gene-engineering. (**A**) Schematic representation of chTCR expression vector. TCR expression cassettes were constructed for TR-CD4, 3B5, and 5B8 CD4^+^ T cell clones and inserted into pMS3 retroviral plasmid vector. LTR: long terminal repeats; *ψ*^+^: extended packaging signal; *SA*: Splice acceptor site from the first exon-intron junction of human elongation factor-1α; hVDJβ: human TCR β chain variable-diverse-joining regions; mCβ: murine TCR β chain constant region; T2A: SGSG-linker connected to the T2A translational skipping sequence; hVJα: human TCR α chain variable-joining regions; mCα: murine TCR α chain constant region. (**B**) Expression of TR-CD4-chTCR. Preactivated human PBMC were infected twice with retroviruses-transducing TR-CD4-chTCR or no vector control (Mock) and 2 days after the second infection, cell surface expression of Vβ5.1 was investigated by flow-cytometry. (**C**) TR-CD4, 3B5, or 5B8-chTCR-transduced PBMC or uninfected (Mock) PBMC were cocultured with DP4^+^NY-ESO-1^+^ SK-MEL-37 or DP4^+^NY-ESO-1^−^ SK-MEL-23 for 24 hours. IFN-γ level in the supernatant was measured by ELISA. Error bars represent the standard deviation from 2 independently infected PBMC from a healthy individual. (**D**) chTCR-transduced or Mock infected PBMC were cocultured with SK-MEL-37 for 6 hours in the presence of Brefeldin A. Expression of IFN-γ was measured by flow-cytometry after staining with cell surface CD8 and CD4 (not shown) and intracellular IFN-γ. Percentage of IFN-γ^+^ cells after coculture with NY-ESO-1^−^ cell lines was less than 2% in both CD4^+^ and CD8^+^ T cells. (**E**) Induction of apoptosis on SK37 by TCR-transduced CD4^+^ T cells. CD8-depleted PBMC were preactivated and transduced with TR-CD4-chTCR as described above. SK37 (1 × 10^5^ cells) were cocultured with or without parental TR-CD4, TR-CD4-chTCR-transduced CD4 or Mock-transduced CD4 at 2 × 10^5^ cells. Apoptotic cell death on SK37 was determined by staining with anti-annexin V antibody at day 1 and day 2. (**F**) SK37 was co-cultured with ESO-CD8 at 1:2 ratio in the presence or absence of indicated numbers of parental TR-CD4 or TR-CD4-chTCR-transduced CD4. Cytotoxicity was evaluated by CFSE-based cytotoxicity assays. All experiments were repeated using PBMC from 2 different healthy individuals with similar results.

Expression cassettes for chimeric human/murine TCR (chTCR) were constructed for TR-CD4 clone as well as 5B8 tumor-recognizing and 3B5 non-tumor-recognizing CD4^+^ T cell clones from our previous study^21^ (Fig. 6A). chTCR is a fusion of human variable region and murine constant region of α and β chains, and is known to avoid mispairing with endogenous human TCR and enhance reactivity of transduced TCR^22^. chTCR gene was retrovirally transduced into PBMC which were preactivated in the presence of IL-2, IL-7 and IL-12. Transduction efficacy was determined to be more than 90% by Vβ5.1 staining for TR-CD4 chTCR-transduced PBMC in 3 healthy individuals (Fig. 6B and data not shown). Although TCR Vβ subtype-specific antibody is not available for 5B8 and 3B5 TCR, we confirmed that 5B8 and 3B5 chTCR-transducing retroviruses induced cell surface CD3 expression on J.RT3-T3.5 TCR β chain-deficient Jurkat cell line (obtained from ATCC) with similar efficiency to that by TR-CD4 chTCR-transducing virus (data not shown).

Direct recognition of NY-ESO-1^+^DP4^+^ SK37 by chTCR-transduced PBMC was tested by measuring IFN-γ levels in the supernatant from coculture of PBMC and SK37. As shown in Fig. 6C, strong recognition of SK37 was exhibited by PBMC when they were transduced with tumor-recognizing TR-CD4 and 5B8 chTCR but not non-tumor-recognizing 3B5 chTCR. Intracellular cytokine staining demonstrated that chTCR-transduced CD4^+^ T cells mainly reacted to SK37 in comparison with much weaker reactivity by CD8^+^ T cells, indicating that our MHC-II-restricted tumor-recognizing TCR are largely CD4-dependent, i.e. co-ligation of CD4 with MHC-II is required for full activation of T cells (Fig. 6D). Next, we assessed the *in vitro* anti-tumor effect of TR-CD4 chTCR-transduced CD4^+^ T cells and compared with that of parental TR-CD4. Even though CD4^+^ T cells were cultured in the presence of IL-12 which strongly promoted IFN-γ-producing type-1 T-cell differentiation, chTCR-expressing CD4^+^ T cells did not exhibit immediate cytotoxicity (data not shown). However, similar to parental TR-CD4, chTCR-expressing CD4^+^ T cells induced apoptosis on SK37 and enhanced ESO-CD8 cytotoxic activity (Fig. 6E,F).

## Discussion

The role of CD4-help in CD8^+^ T cell priming, effector function, and memory responses has been extensively studied in recent years. Although provision of CD4-help classically requires APCs via CD40-CD40L^16^ and CD27-CD70 interactions^17^, our current study provides evidence that direct cognate interaction between a subset of human CD4^+^ Th1 (TR-CD4) cells and cancer cells potently provide help to CD8^+^ T cells in an APC-independent fashion. Upon directly recognizing cancer cells, TR-CD4 express an array of effector molecules, including IL-2, IFN-γ, TNF-α, perforin and granzyme, which play critical roles in inhibiting cancer cell growth and orchestrating anti-tumor immune responses. Although CD4^+^ T cells could acquire cytotoxic activity such as cytotoxic CD4^+^ T cells^23^, our TR-CD4 did not show significant cytotoxicity *in vitro*. However, they significantly delayed cancer cell growth *in vitro* and *in vivo*, and enhanced *in vitro* cytolytic function of CD8^+^ T cells without requiring APCs. In the presence of TR-CD4, tumor-activated CD8^+^ T cells were endowed with abilities to efficiently proliferate, survive, and upregulate central memory markers. IL-2 produced from TR-CD4 played a role in the enhancement of functions of CD8^+^ T cells because addition of supernatant of activated TR-CD4 or recombinant IL-2 partially reproduced the enhancement (Fig. 4E,F). Recently, it was reported that direct cellular interaction between CD4^+^ and CD8^+^ T cells enhances the functions of CD8^+^ T cells^24^,^25^. Because TR-CD4 and CD8^+^ T cells formed clusters with cancer cells when they were cocultured (Fig. 4A), it is possible that TR-CD4 enhance the function of CD8^+^ T cells by direct cellular interaction in the cluster. In contrast to our *in vitro* experiments, there are limitations of our *in vivo* therapeutic model (Fig. 3D,F). The model did not clearly demonstrate synergistic therapeutic effects by TR-CD4 and CD8^+^ T cells through TR-CD4-mediated enhancement of CD8^+^ T-cell functions. It is also important to note that our *in vitro* and *in vivo* experiments in this study did not investigate the role of APCs. Establishment of *in vitro* co-culture systems with APCs or more physiological *in vivo* animal models using humanized or HLA-transgenic animals will be required to elucidate the role of APCs and TR-CD4-mediated “CD4-help” to CD8^+^ T cells.

TR-CD4 efficiently inhibited *in vivo* tumor growth to a level that is comparable to that obtained with strongly cytotoxic NY-ESO-1-specific CD8^+^ T cells. As one of the potential mechanisms, we demonstrated that TR-CD4-derived IFN-γ induced long-term growth arrest of cancer cells. It is known that IFN-γ mediates growth arrest in a STAT-1-dependent manner^26^. More recently, Braumuller *et al*. reported that the combination of Th1-derived cytokines, IFN-γ and TNF-α, induces G0/G1 arrest and permanent senescence in pancreatic β-cancer cell^14^. In our experiments using SK37 melanoma cell line, IFN-γ alone or IFN-γ and TNF-α treatment did not induce senescence, but these cytokines induced G2/M arrest, potentially because of the different cancer cell line used or marginal expression of TNF-α receptor on SK37. Because many human cancers including melanoma and ovarian cancer are IFN-γ and TNF-α-sensitive^14^, it is possible that TR-CD4 will show additional anti-tumor effects against other cancer cell lines that co-express receptors for IFN-γ and TNF-α. Although most CD4^+^ T cells require antigen cross-presentation by APCs to produce Th1 cytokines, our observations indicated that TR-CD4 can bypass the requirement for APCs and directly delay cancer cell growth.

Because of their ability to directly recognize tumor targets, the TR-CD4 in this study is unique and distinct from conventional CD4^+^ T cells that require antigen cross-presentation by APCs for activation. Since APCs within the tumor microenvironment are frequently suppressive, activation of conventional non-tumor-recognizing CD4^+^ T cells and therefore the provision of CD4-help may be limited at the local tumor site. Therefore, the ability of TR-CD4 to directly recognize cancer cells is especially important for the provision of CD4-help in the tumor microenvironment and may thereby lead to durable anti-tumor responses. The patient from whom TR-CD4 and NTR-CD4 were established showed no detectable NY-ESO-1 peptide-reactive CD4^+^ T cells before NY-ESO-1_157–170_ peptide vaccination. After vaccination, peptide-specific CD4^+^ T cells became detectable and a subset of these vaccine-induced CD4^+^ T cells showed tumor-recognizing ability. Since several solid tumors (e.g. ovarian cancer and melanoma) constitutively express or are induced to express MHC-II upon encountering IFN-γ and thus become direct targets of TR-CD4, the generation of effective TR-CD4 responses has significant therapeutic potential and broad clinical relevance^27^,^28^. In support of this notion, we have previously demonstrated that TR-CD4 produced high amounts of IFN-γ upon stimulation with single cell suspensions of NY-ESO-1^+^ malignant tissues freshly isolated from ovarian cancer patients^13^.

The mechanism(s) for the differential ability of TR-CD4 and NTR-CD4 to directly recognize tumor is still unknown. CD4^+^ T cell subset that has the unique property of antigen recognition has been reported by Mohan *et al*. in autoimmune diabetic mice of the nonobese diabetic (NOD) strain^29^. The study identified two types of insulin B:9–23 peptide-specific CD4^+^ T cells; that differ in the recognition of exogenous protein and the ability to induce insulitis because of the recognition of different registers of the peptide on MHC-II. Similarly, difference between TR-CD4 and NTR-CD4 in the direct recognition of cancer cells could be explained, to a large degree, by the slight difference in TCR-peptide/MHC configuration.

TR-CD4 was detected only in 1 out of 11 patients after the peptide vaccination while conventional NTR-CD4 was induced in all patients^21^, indicating that precursor frequency of TR-CD4 is much lower than NTR-CD4. Therefore, the development of novel vaccine approaches to selectively expand TR-CD4; or gene-engineering to generate TR-CD4 for adoptive T cell therapy will be highly desirable. Adoptive transfer of tumor-specific T cells that were generated by *in vitro* stimulation or genetic engineering has proven to be effective in mediating tumor regression in humans^20^,^30^,^31^,^32^. Recently, feasibility and efficacy of adoptively transferred MHC-I-restricted TCR gene-engineered T cells for the treatment of cancer patients have been demonstrated in clinical trials including those testing A2-restricted NY-ESO-1-specific TCR-transduced T cells^18^,^33^. However, it was found that adoptive transfer of MHC-I-restricted, TCR-transduced cells alone may not be sufficient for complete elimination of cancer cells, potentially indicating insufficient persistence or tumor-mediated immune suppression at the tumor sites^19^,^34^. In addition, infusion of NY-ESO-1-specific CD4^+^ T cells, that were NY-ESO-1_157–170_-reactive and DP4-restricted, resulted in complete remission in one patient^20^. In a murine model, adoptive transfer of MHC-II-restricted TRP-specific TCR-engineered T cells induced tumor regression while MHC-I restricted TCR-transduced CD8^+^ T cells only delayed tumor growth^35^. In addition, chimeric antigen-receptor transduced CD4^+^ T cells showed potent anti-tumor immune responses^36^. These findings indicate that tumor antigen-specific CD4^+^ T cells could be potent anti-tumor effector cells if they are able to directly recognize cancer cells. In our study, we demonstrated that large numbers of CD4^+^ T cells that strongly react against tumor targets in a MHC-II-restricted manner can be generated by gene-engineering with TCR from tumor-recognizing CD4^+^ T cell clones. A future approach is to test TCR gene-engineered tumor-recognizing CD4^+^ T cells for their ability to potently mediate anti-tumor effects alone or in combination with MHC-I-restricted TCR-transduced T cells. These previous findings, coupled with our demonstration that MHC-II-expressing cancer cells can be efficiently recognized by TR-CD4, which in turn augments anti-tumor CD8^+^ T-cell response, indicate that immunotherapy focusing on tumor-recognizing CD4^+^ T cells could be a promising strategy for effective eradication of tumors.

## Methods

### NY-ESO-1 specific T cells and cell lines

NY-ESO-1 specific T cell clones were established as described previously. Briefly, NY-ESO-1-specific CD4^+^ and CD8^+^ T cells were amplified by *in vitro* presensitization from patients who received NY-ESO-1 vaccination^12^,^37^. PBMC of epithelial ovarian cancer patients were obtained with informed consent at Roswell Park Cancer Institute in accordance with an approved protocol from the institutional review board. A2-restricted NY-ESO-1_157–165_-specific CD8^+^ T cells were isolated by a FACSAria instrument (BD Biosciences) using HLA-A2/NY-ESO-1_157–165_ tetramer. DP4-restricted NY-ESO-1_157–170_-specific CD4^+^ T cells were restimulated with DP4-binding peptide for 4 hours and isolated by a FACSAria instrument by gating on CD40L^+^ cells or IFN-γ^+^ cells following staining with IFN-γ secretion assay kit according to manufacturer’s instruction (Miltenyi Biotec)^38^. CD4^+^ T cells were cloned by limiting dilution and periodic phytohemagglutinin (PHA; Remel) stimulations in the presence of feeder cells (irradiated allogeneic PBMC) and IL-2 (Roche Molecular Biochemicals). Cells were cultured in RPMI1640 medium supplemented with 10% FBS, penicillin, streptomycin and L-glutamine.

### *In vitro* cytotoxicity assay

*In vitro* cytotoxiocity assay was performed using CFSE-based labeling^6^. SK37 or peptide-pulsed SK29 were labeled with 0.5 μM CFSE, whereas peptide-unpulsed SK29 was labeled with 5 μM CFSE. In some experiments, cancer cells were treated with 20 μg/ml Fas-ligand antibodies (eBiosciences) for 1 hour before addition to T cells. Fas-ligand antibody was also present during co-culture. 2 U/ml of recombinant IL-2, combination of 10 μg/ml anti-CD25 (IL-2Rα) antibody (BD Bioscience) and anti-IL-2 antibody (eBioscience), 0.1 nM concanamycin A (Sigma-Aldrich) or 100 μM Z-AAD-CMK (Enzo Biochem, Inc.) were added at the initiation of T cell culture. Cells were incubated in 5 mL round-bottom tubes for 14–16 hours. Cancer cells were harvested by treatment with trypsin/EDTA and analyzed for CFSE staining levels by FACSCalibur flow cytometer (BD Biosciences) to enumerate the percentages of SK37 and SK29. Acquisition data were analyzed by FCS Express Version 3 software (De Novo Software) or FlowJo software. Cytotoxicity was calculated using the following formula: % cytotoxicity = 100 × {1−(%SK29/%SK37)_without T cells_/(%SK29/%SK37)_with T cells_}.

### Cell proliferation

To assess the effect of cytokines or T cells on cancer cell proliferation, 1 × 10^4^ SK37 were co-incubated with or without 100 ng/ml IFN-γ and/or 10 ng/ml TNF-α, 2 × 10^5^ CD4^+^ T cells, or 2 × 10^5^ CD8^+^ T cells in a 24-well culture plate. To determine effector molecules in TR-CD4-mediated cell growth arrest, 1 × 10^5^ SK37 were co-cultured with 2 × 10^5^ TR-CD4 in the presence or absence of 10 μg/ml anti-IFN-γ (eBiosciences) plus 10 μg/ml anti-IFN-γ receptor (BD Biosciences) mAbs, 10 μg/ml anti-TNF-α mAb (eBiosciences), the combination of these antibodies, 0.1 nM concanamycin A or 100 μM Z-AAD-CMK. Cell proliferation of SK37 was determined by trypan blue exclusion or MTT assays.

### Cell cycle analysis

SK37 (2 × 10^4^) with or without 100 ng/ml IFN-γ and 10 ng/ml TNF-α, or 5 × 10^4^ SK37 and 2 × 10^5^ TR-CD4 in the presence or absence of anti-IFN-γ or anti-IFN-γ/anti-TNF-α mAbs were cultured in a 24-well culture plate. At day 3, 10 μM of BrdU were added into the culture for 4 hours. Cells were harvested and stained using BrdU staining kit (BD Biosciences) according to manufacturer’s instruction.

### *In vivo* xenograft mouse model

Animal experiments were performed in accordance with an approved protocol from the Institutional Animal Care and Use Committee (Roswell Park Cancer Institute). Cells were washed twice with PBS, resuspended in PBS, and incubated on ice just before injection. 1 × 10^6^ cancer cells mixed with or without 2 × 10^6^ CD8^+^ T cells and/or 2 × 10^6^ CD4^+^ T cells were intradermally injected into the flank of SCID mice. Tumor size was measured by calipers every 2–3 days following injection. Tumor volume was calculated by following formula; 0.5 × (length × width × height). Tumor tissue was excised and weighed when the tumor volume of any mice reached 1,000 mm^3^.

### Microscopic analysis

Cells were labeled with PKH67 (for CD8^+^ T cells, Sigma), PKH26 (for CD4^+^ T cells, Sigma) or CellBrite blue cytoplasmic membrane staining kit (for SK37, Biotium, Inc.) according to the manufacturer’s instruction. SK37 (2.5 × 10^5^) were co-cultured with CD4^+^ T cells (5 × 10^5^) and/or CD8^+^ T cells (5 × 10^5^) in a 24-well culture plate. Pictures of cells were taken by a fluorescent microscope (SPOT Imaging Solutions) at 10 × 20 magnification. Cell motion was monitored every 5 minutes for 16 hours at 10 × 10 magnification by Leica Live Cell Imaging Systems under the controlled temperature and atmosphere.

### Measurement of cytokines

CD8^+^ T cells (5 × 10^4^), CD4^+^ T cells (5 × 10^4^) or the combination of these cells were cultured with SK37 (2.5 × 10^4^) in a 96-well culture plate. The culture supernatant was collected day 1 to day 4 after the culture and stored at −20 °C until measurement of cytokines by ELISA according to manufacturer’s instruction. ELISA kits for IL-2, IFN-γ and TNF-α were obtained from eBioscience, and the kits for perforin and granzyme B were obtained from Mabtech, Inc.

### Phenotypic and functional analysis

T cell and cancer cell phenotype was analyzed by using antibodies for cell surface molecules. Granzyme B, perforin and NY-ESO-1 expression in cytoplasmic were analyzed following fixing and permeabilization (Invitrogen-CALTAG). Antibodies for cell surface molecules, perforin and annexin-V were purchased from BD Biosciences except for granzyme B antibody (Diagnostika).

### Generation of NY-ESO-1 and CIITA co-expressing ovarian cancer cell lines

HLA DPB1 genotype for ovarian cancer cell lines was determined by the SSP-PCR method^39^. Protein-coding regions of NY-ESO-1 and Sp17 were PCR amplified from cDNA of SK37 and cloned into the first multiple cloning site in a pQCXIX self-inactivating retroviral vector (Clontech). CIITA-coding DNA was also amplified from SK37 cDNA and cloned into the second multiple cloning site in the same vector following the internal ribosomal entry site (IRES). Resulting pQC-NY-ESO-1-IRES-CIITA or pQC-Sp17-IRES-CIITA plasmid was co-transfected together with a pVSV-G plasmid (Clontech) into a GP2-293 packaging cell line (Clontech) using Lipofectamine 2000 reagent (Invitrogen). Retroviral supernatant was added on DP4^+^ ovarian cancer cell lines (OV2774 and OVCAR-5) in the presence of 8 μg/ml polybrene (Sigma). Five to 10 days after infection, cells were stained with anti-HLA-DR antibody and HLA-DR^+^ cells were sorted by a FACSAria instrument.

### Retroviral transduction of TCR gene

Full-length TCR α and β chain genes of CD4^+^ T cell clones (TR-CD4, 5B8, and 3B5) were cloned by 5′ RACE PCR using Smarter RACE cDNA Amplification and Advantage 2 PCR kits (Clontech) using gene-specific primers for the 3′ untranslated region for human α, β1, β2 chains^40^. Constant regions for murine TCR α and β chain genes were cloned from cDNA of the thymus from a C57BL/6 mouse using Q5 DNA Polymerase (New England Biolabs). The variable region of human TCR α or β chain gene was fused to the corresponding murine TCR constant region by the recombinant PCR method using Q5 DNA Polymerase. Human/murine chimeric TCR β and α chain genes were connected via SGSG-linker-T2A sequence using the recombinant PCR method^41^. The expression cassettes were inserted into pMS3 retroviral vector^41^. Retroviruses-carrying chimeric TCR gene were transiently produced from a GP2-293 packaging cell line (Clontech) by transfection of pMS3 and pVSV-G (Clontech) plasmids using Lipofectamine 2000 reagent (Invitrogen) and were added on PG13 packaging cell line (ATCC). High-titer retrovirus-producing PG13 clones were obtained by limiting dilution and by screening by infection into J.RT3-T3.5 cell line (ATCC) followed by cell surface staining of CD3 (BioLegend).

PBMC from healthy individuals, that were obtained from the Donor Center at Roswell Park Cancer Institute, were preactivated for 2 days in the presence of 10 μg/ml PHA^15^ (Remel), 10 U/ml recombinant human IL-2 (Roche), 10 ng/ml recombinant human IL-7 (R&D systems), and 10 ng/ml recombinant human IL-12 (Peprotech). Preactivated PBMC were infected on a 10 μg/ml RetroNectin (TaKaRa Bio-Clontech) and 5 μg/ml anti-CD3 mAb (OKT3; eBioscience)-coated plate by the RetroNectin-Bound Virus (RBV) method as suggested by the manufacturer in the presence of IL-2, IL-7 and IL-12. Infection was repeated similarly but without anti-CD3 mAb 24 hours later, and expanded for 5–7 days in the presence of IL-2 and IL-7 but without IL-12. Transduction efficacy was measured for TR-CD4-chTCR by staining with anti-human TCR Vβ5.1 mAb (eBioscience). In some experiments, CD8^+^ T cells were depleted from PBMC by magnetic beads (Invitrogen) prior to activation with PHA. After expansion and retroviral transduction, purity of CD4^+^ T cells as determined by flow cytometry was greater than 95%.

### Statistical analyses

Data are shown as means and standard deviations except for *in vivo* tumor growth (means and standard error of the mean). *P* values of less than 0.05 were considered statistically significant by unpaired Student’s *t*-test. All statistical analyses were performed using Prism 5 software (GraphPad Software).

## Additional Information

**How to cite this article**: Matsuzaki, J. *et al*. Direct tumor recognition by a human CD4^+^ T-cell subset potently mediates tumor growth inhibition and orchestrates anti-tumor immune responses. *Sci. Rep*. **5**, 14896; doi: 10.1038/srep14896 (2015).

## Supplementary Material

Supplementary Information

Supplementary Movie S1
