# Supplementary material for: Direct tumor recognition by a human CD4+ T-cell subset potently mediates tumor growth inhibition and orchestrates anti-tumor immune responses
Source: Sci Rep. 2015 Oct 8;5:14896. doi: 10.1038/srep14896 (PMC4597193; doi:10.1038/srep14896)
Supplement: Supplementary Information [file srep14896-s1.pdf]

Supplementary information

Direct tumor recognition by a human $CD4^{+}$ T-cell subset potently mediates tumor growth inhibition and orchestrates anti-tumor immune responses

Junko Matsuzaki, Takemasa Tsuji, Immanuel F. Luescher, Hiroshi Shiku, Junichi Mineno, Sachiko Okamoto, Lloyd J. Old, Protul Shrikant, Sacha Gnjatic, and Kunle Odunsi

Supplementary Movie S1. Live cell imaging for interaction of cancer cells and T cells. SK37 (blue), ESO-CD8 (red) and TR-CD4 (green) were co-cultured following labeling with fluorescent dyes. Starting from 4 hours of co-culture, pictures were taken every 5 minutes for 16 hours. Scale bar indicates 100 μm with 20 μm minor divisions.

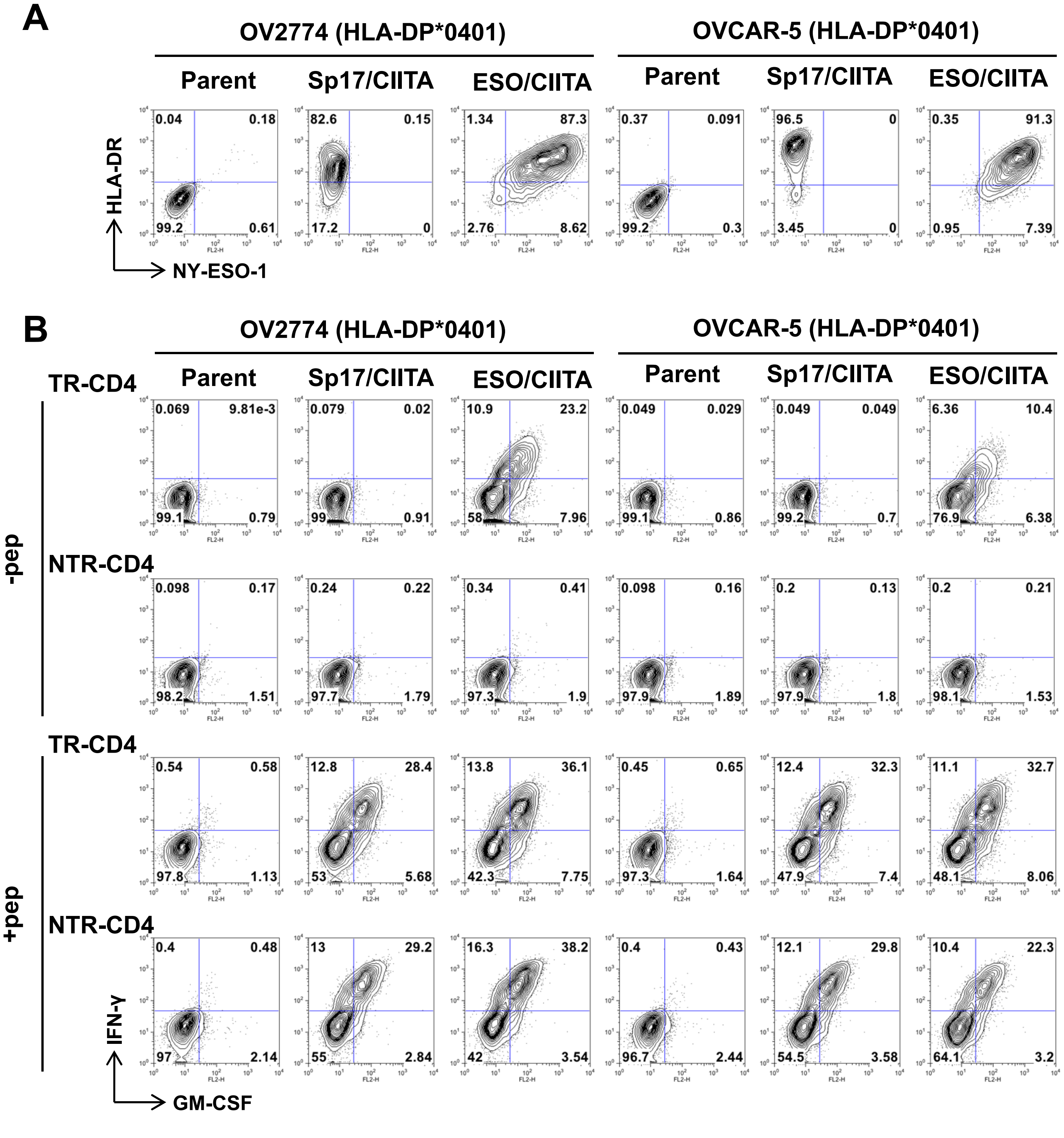

Supplementary Figure S1. Reactivity of TR-CD4 and NTR-CD4 against ovarian cancer cell lines co-transduced with NY-ESO-1 and class II trans-activator. (A) Generating NY-ESO-1^{+}HLA-DR^{+} ovarian cancer cell lines. Class II trans-activator (CIITA) along with NY-ESO-1(ESO) or sperm protein (Sp17) was retrovirally co-transduced in HLA-DP*04^{+} OV2774 and OVCAR-5 ovarian cancer cell lines. NY-ESO-1 and HLA-DR expression were examined in parental or gene-engineered cells after sorting of HLA-DR^{high} cells by FACSAria. (B) Reactivity of TR-CD4 and NTR-CD4 against ovarian cancer cell lines. IFN-γ and GM-CSF production in TR-CD4 or NTR-CD4 was determined by intracelluar cytokine staining following 6 hours stimulation with NY-ESO-1$_{157\text{-}170}$-peptide unpulsed or pulsed indicated cell lines.

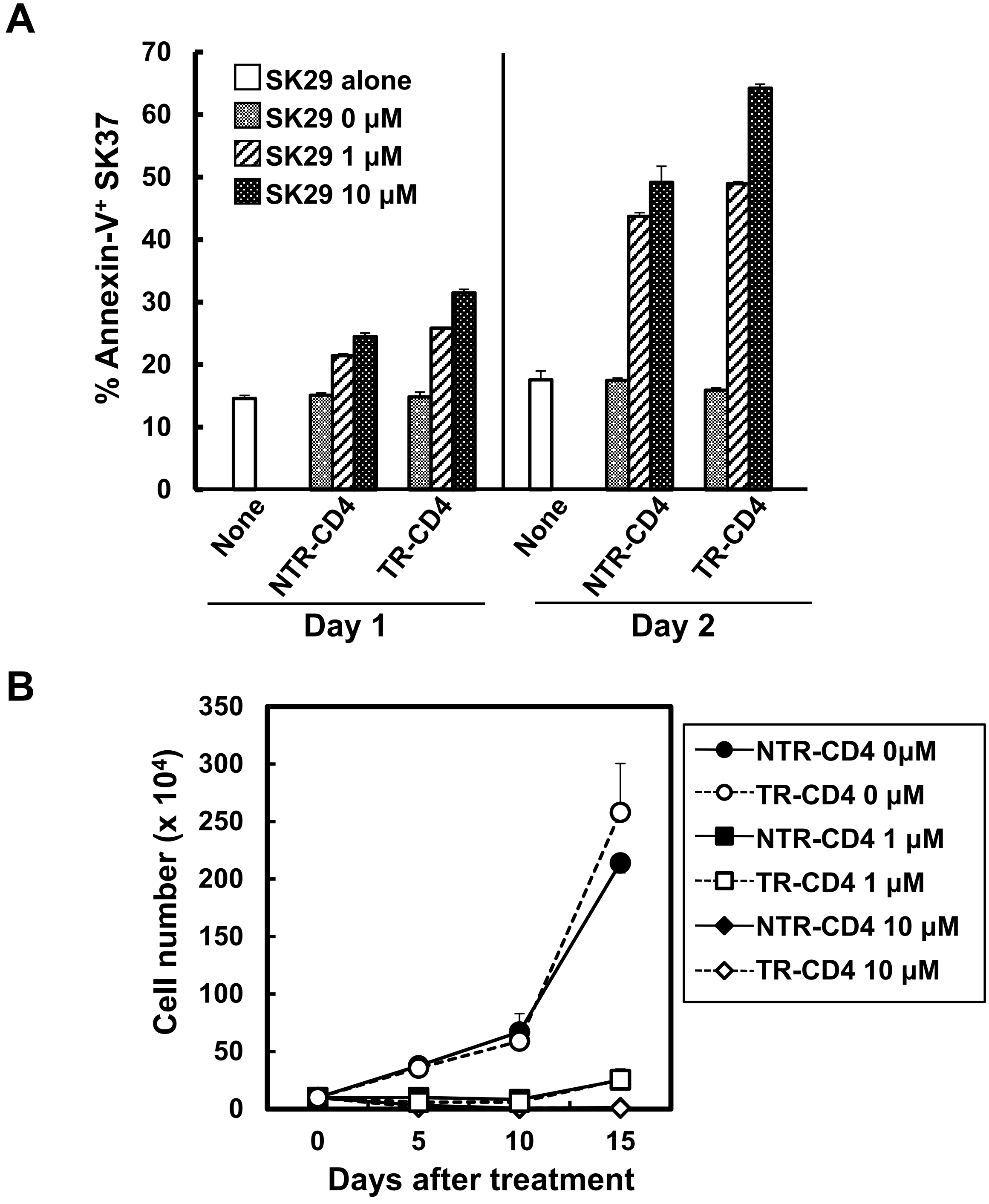

Supplementary Figure S2. Growth arrest of peptide-pulsed SK29 by NTR-CD4 and TR-CD4. SK29 (1×10^5 cells) pulsed with or without indicated concentration of NY-ESO-$1_{157\text{-}170}$ peptide were coculture with 2×10^5 NTR-CD4 or TR-CD4. (A) At day 1 and day 2, cells were harvested and apoptotic cell death was investigated by staining with anti-annexin-V antibody. (B) SK29 cell growth in the presence of NTR-CD4 or TR-CD4. Non-adherent cells were removed by repeated rinses using culture medium and adherent SK29 were further cultured for 10 days. Cell numbers were determined by trypan blue exclusion assays.

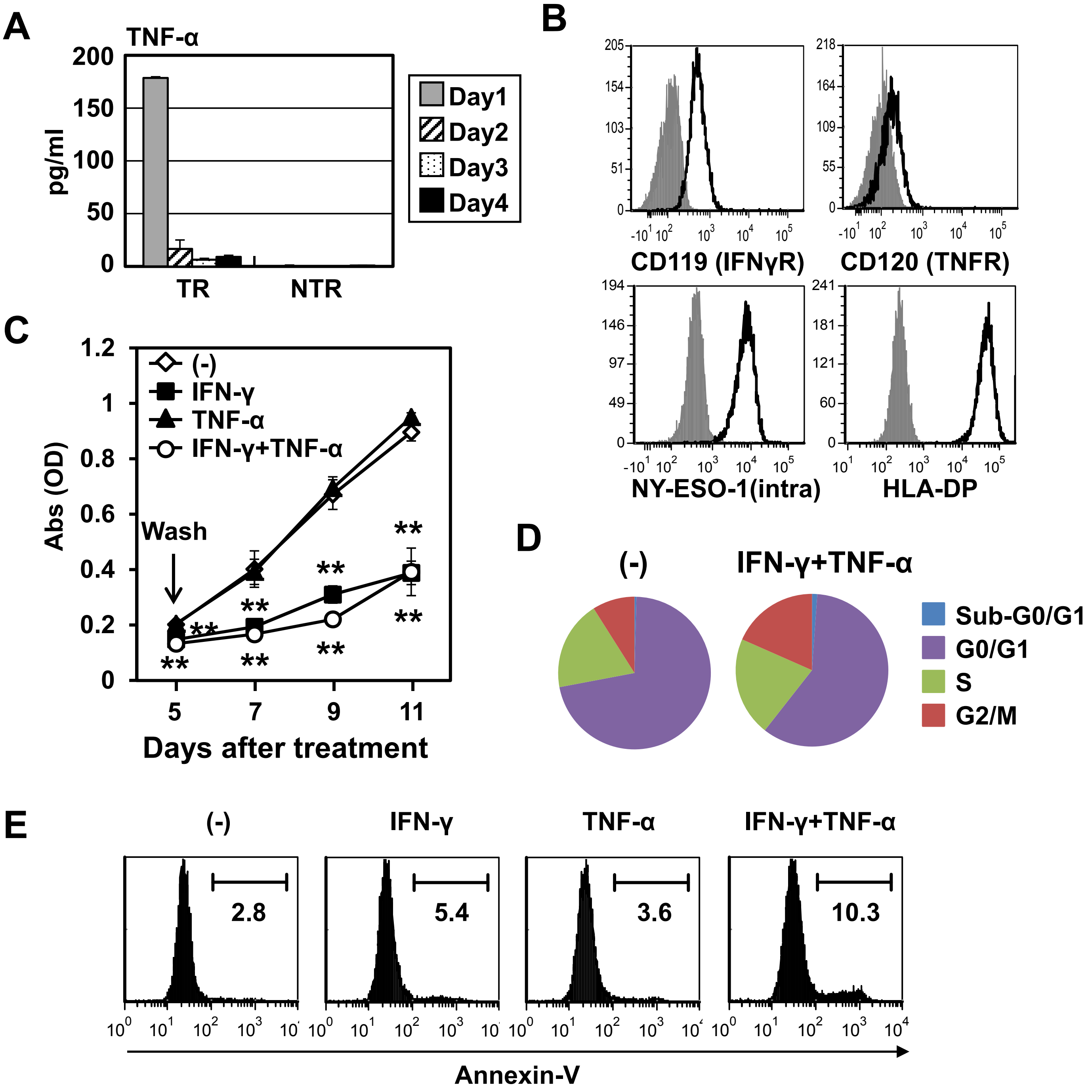

Supplementary Figure S3. Effect of IFN-γ and TNF-α on SK37. (A) TR-CD4 and NTR-CD4 were co-cultured with SK37 and supernatant was collected every 24 hours for 4 days. TNF-α level in the supernatant measured by ELISA is shown. Background production against unpulsed SK29 was below the detection limit (4 pg/ml). (B) Characterization of SK37. SK37 was stained with antibodies against indicated molecules. Fluorescent signals were measured by flow cytometry. Shaded histograms indicate background staining by isotype controls. (C, D) SK37 was cultured with 100 ng/ml IFN-γ and/or 10 ng/ml TNF-α. (C) Cells were rinsed with culture medium three times to remove cytokines at day 5. Cell proliferation was determined by MTT assay at indicated time points after the treatment. (D) Cell cycle was examined by BrdU incorporation assay at day 3 after the culture. (E) SK37 was cultured with 5,000 pg/ml IFN-γ and/or 500 pg/ml TNF-α for 2 days. These concentrations of cytokines were determined based on the cytokine levels in the supernatant of TR-CD4 after stimulation with SK37. Apoptotic cell death was investigated by staining with anti-annexin-V antibody. ***p* < 0.01 as compared with control, Student's t-test.

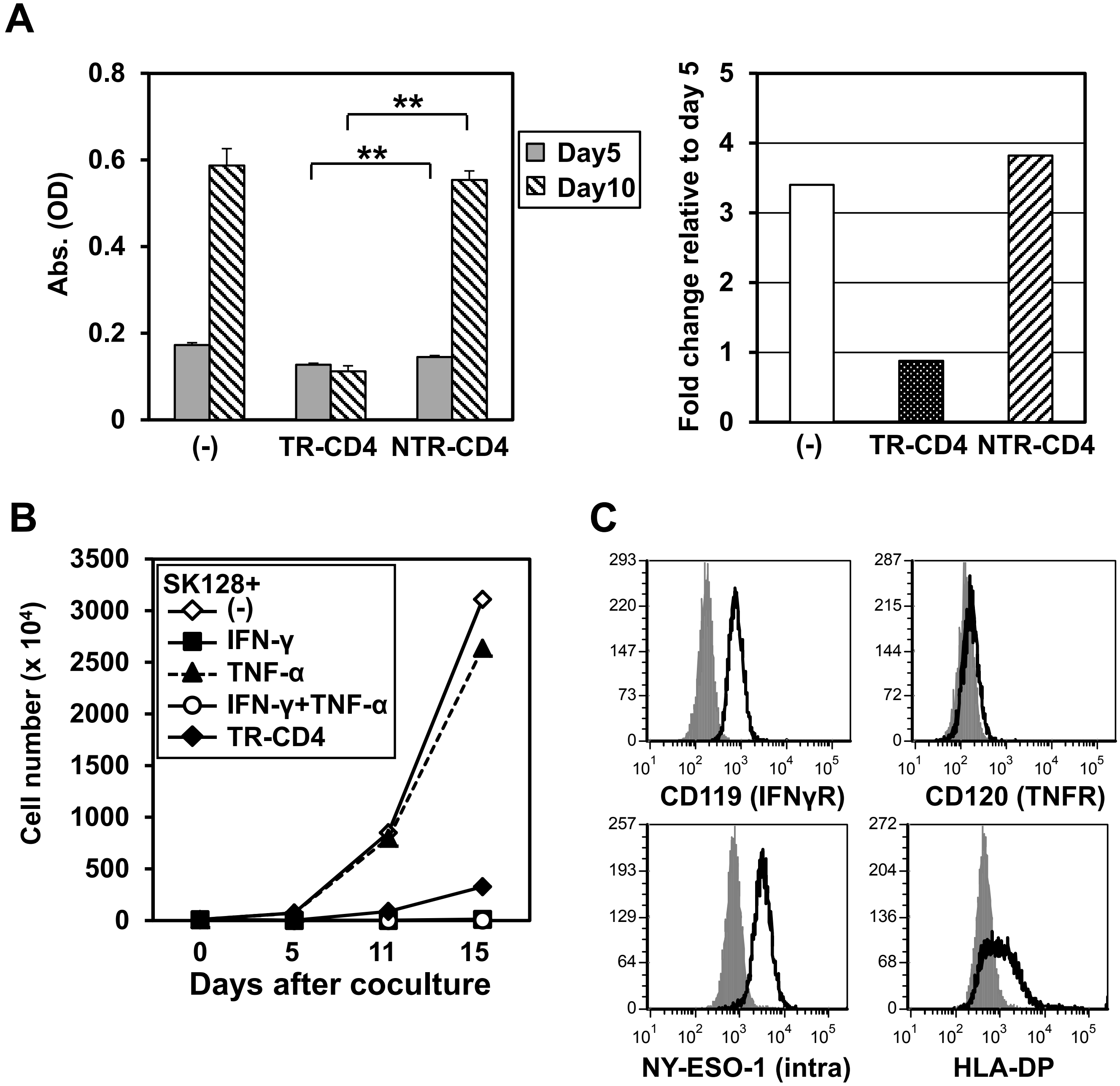

Supplementary Figure S4. SK-MEL-128 cell growth inhibition by TR-CD4. (A) One hundred SK-MEL-128 (SK128) were cocultured with 2 × 10^4 $CD4^+$ T cells for 5 days, and then non-adherent cells were removed by rinsing with the culture medium three times. Cell number was determined by MTT assay at indicated time points after the culture. Fold change indicates ratio of absorbance at day 10 compared with absorbance at day 5 (right). (B) SK128 was co-cultured with or without TR-CD4 or cytokines for 5 days. Non-adherent cells were removed by repeated rinses using culture medium and adherent SK128 were further cultured for 10 days. Cell numbers were determined by trypan blue exclusion assays. (C) Characterization of SK128. SK128 was stained with antibodies against indicated molecules. Fluorescent signals were measured by flow cytometry. Shaded histograms indicate background staining by isotype controls. ***p* < 0.01 by Student's t-test.

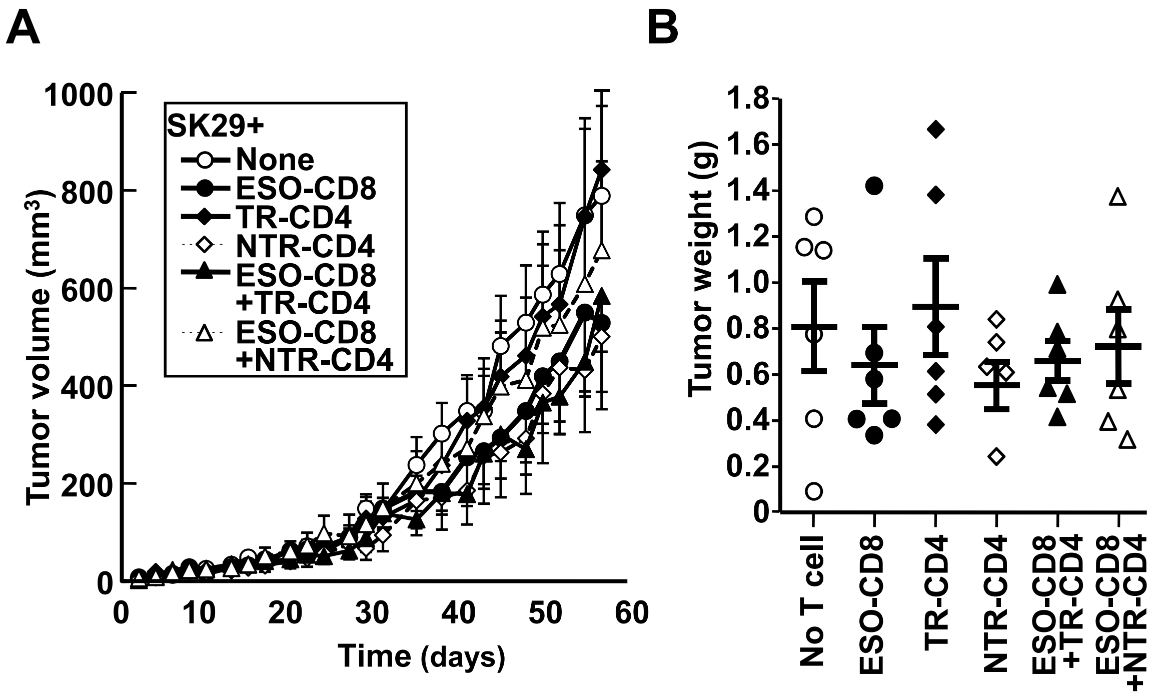

Supplemental Figure S5. Effect of NY-ESO-1-specific T cells on *in vivo* growth of NY-ESO-1-negative melanoma. HLA-A2$^+$HLA-DP4$^+$NY-ESO-1$^-$ SK29 was inoculated into SCID mice (n=6 per group) together with or without ESO-CD8 and/or TR-CD4 or NTR-CD4. (A) Tumor size was measured by caliper every 2-3 days. (B) Tumor was excised and weighed at day 57 after inoculation. Results are shown mean + SEM.

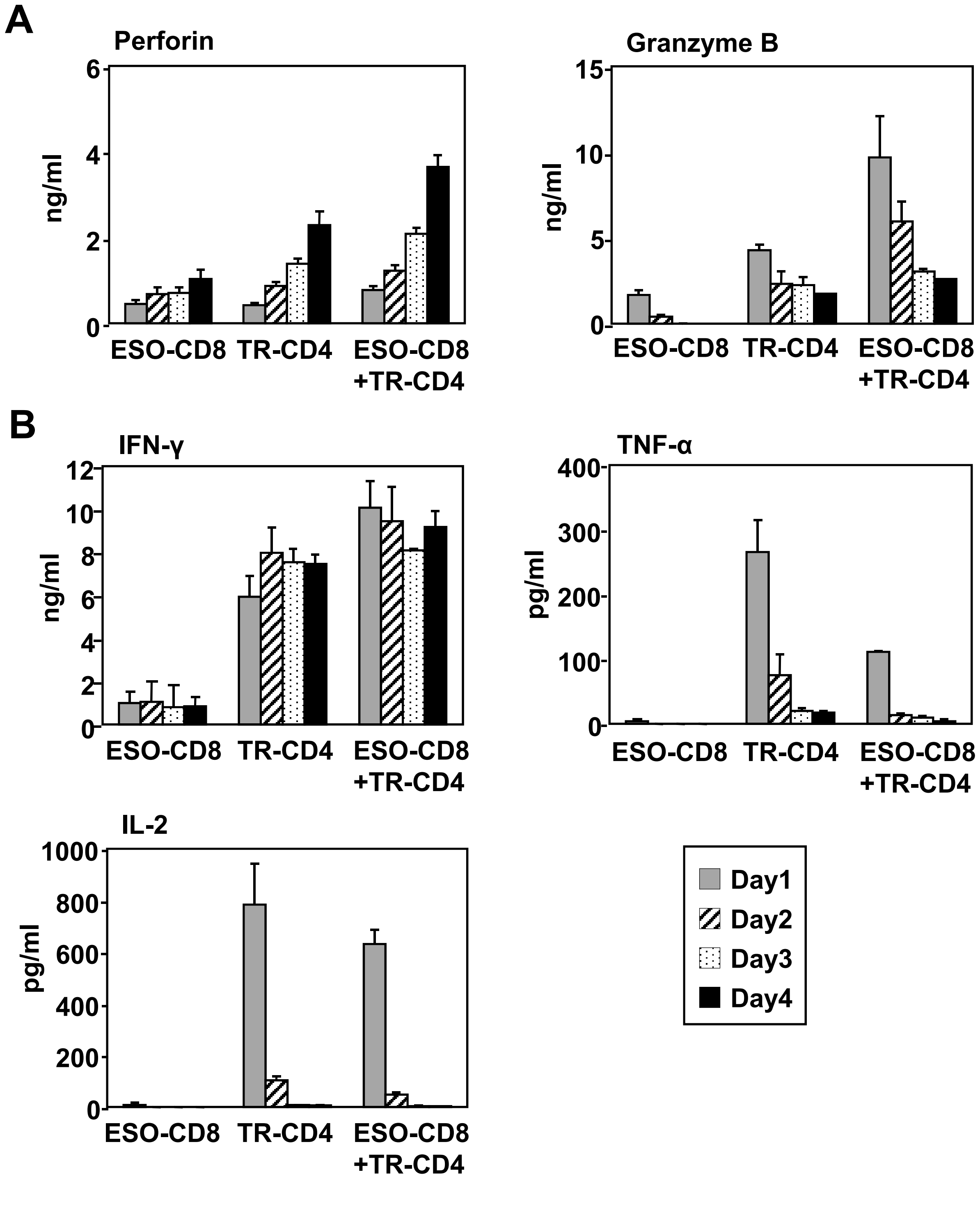

Supplementary Figure S6. Cytokine production from ESO-CD8 and TR-CD4 stimulated by SK37. ESO-CD8 or TR-CD4 was co-cultured with SK37 and supernatant was collected every 24 hours for 4 days. (A) Perforin and granzyme B, (B) IFN-γ, TNF-α and IL-2 levels in the supernatant measured by ELISA are shown.

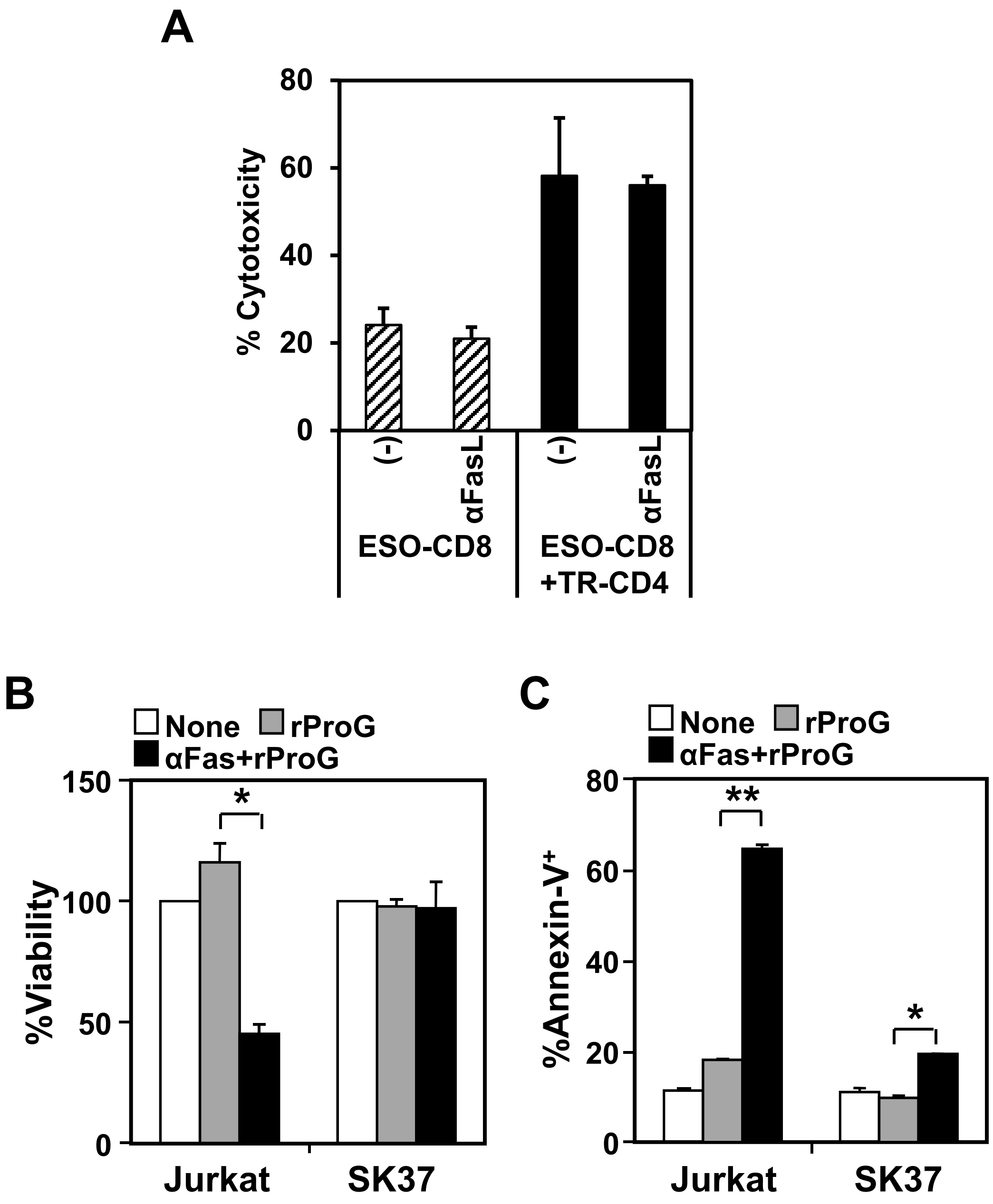

Supplementary Figure S7. Resistance of SK37 against Fas-FasL-mediated killing. (A) Effect of anti-FasL antibody on CD8+ T cell cytotoxicity. Cytotoxic activity of ESO-CD8 against SK37 in the presence or absence of TR-CD4 was tested by CFSE-based cytotoxicity assays. For blockade of Fas-FasL interaction, 20 μg/ml anti-FasL antibody (αFasL) was added to SK37 1 hour before co-culture and also during assays. (B, C) SK37 and Jurkat cells were cultured with or without recombinant Protein G (rProG) and/or anti-Fas antibody (αFas). (B) Cell viability was determined by the MTT assay, and (C) apoptotic cell death was measured by annexin-V staining. *$p < 0.05$, **$p < 0.01$ by Student's *t*-test.
